# miR‐34a‐5p Attenuates EMT through targeting SMAD4 in silica‐induced pulmonary fibrosis

**DOI:** 10.1111/jcmm.15853

**Published:** 2020-09-14

**Authors:** Yuanmeng Qi, Ahui Zhao, Peiyan Yang, Luheng Jin, Changfu Hao

**Affiliations:** ^1^ School of public health Zhengzhou University Henan China

**Keywords:** epithelial‐mesenchymal transition, miRNA, silicosis, SMAD4

## Abstract

Silicosis is an incurable occupational disease, and its pathological feature is diffuse pulmonary fibrosis. Pulmonary epithelial‐mesenchymal transition (EMT) is one of the important events in the pathogenesis of silicosis. Previous studies found that abnormal expression of various microRNAs (miRNAs) involved in the development of lung fibrosis. However, their roles in silicosis have not been elucidated. To research the biological effects of miR‐34a in EMT process in silica‐induced lung fibrosis, we established the silicosis model in mouse and miR‐34a intervention in a cell model of TGF‐β1 stimulated lung epithelial cells (A549). The results showed that miR‐34a expression was down‐regulated in the fibrotic lung tissue after silica treatment, and it was similarly expressed in A549 cells stimulated by TGF‐β1. Meanwhile, silica‐induced EMT process can increase expression of two mesenchymal markers, α‐SMA and vimentin. Furthermore, overexpression miR‐34a markedly inhibited EMT stimulated by TGF‐β1. Mechanistically, SMAD4 was identified as the target of miR‐34a. SMAD4 levels decreased in mRNA and protein levels in A549 cells upon miR‐34a overexpression. In addition, the knockdown of SMAD4 blocked the EMT process. Taken together, miR‐34a regulated EMT, which might be partially realized by targeting SMAD4. Our data might provide new insight into treatment targets for silica‐induced pulmonary fibrosis.

## 1 INTRODUCTION

Silicosis is a common interstitial pulmonary fibrosis (IPF) caused by inhalation of silica for a long time in crystalline form or silicon dioxide.^1^ Pathologically, epithelial‐mesenchymal transition (EMT) and extracellular matrix (ECM) excessive deposition are involved in the development of silicosis.^2^ Previous study^3^ has shown that EMT plays an important role in the progression of lung fibrosis. Moreover, TGF‐β1 is known to be a major cytokine that induces EMT. Emerging evidence has confirmed that the activated TGF‐β signalling pathway is closely related to silicosis.^4^ Nowadays, studies have shown that SMAD4, a member of SMAD family, as a key step in the TGF‐β signalling pathway, plays an important role in EMT.^5^ miRNAs dysregulation has been showed to be significantly associated with pulmonary fibrosis, such as EMT.^6^, ^7^ A recent research has shown that miR‐34a inhibits liver fibrosis by targeting SMAD4.^8^ In addition, no previous study has explored the relationship for SMAD4 and miR‐34a in silicosis. In this study, we focused on exploring the role of SMAD4 and miR‐34a in mouse silicosis fibrosis and the EMT induced by TGF‐β1 in vitro.

## 2 MATERIALS AND METHODS

### 2.1 Animals

Male C57BL/6 mice (5‐6 weeks of age, 16‐18 g) were purchased from Vital River Laboratory Animal Technology. Animal feeding conditions were proved by Use Committee of Zhengzhou University.

### 2.2 Silica particles

The acquisition of silica particles and their size distribution have been shown in detail in the supplementary materials.

### 2.3 Mouse silicosis model and experiment design

The mice were randomly means into experimental groups at different time points (inducing 1, 14, 28 and 56 days) and corresponding normal saline control group. The pulmonary fibrosis model of mice was established by intratracheal infusion of 50 μL SiO_2_ suspension (50 mg/mL) or volume saline.

### 2.4 Histopathology analysis

See supplementary materials.

### 2.5 Cell cultures and Cell transfection

See supplementary materials.

### 2.6 RT‐PCR, Western blot and Dual‐luciferase reported gene assays

See supplementary materials.

### 2.7 Immunofluorescence assay

See supplementary materials.

### 2.8 Statistical analysis

See supplementary materials.

## 3 RESULTS

### 3.1 Expression of miR‐34a‐5p and SMAD4 in lung tissues during silica‐induced pulmonary fibrosis

**FIGURE 1:**
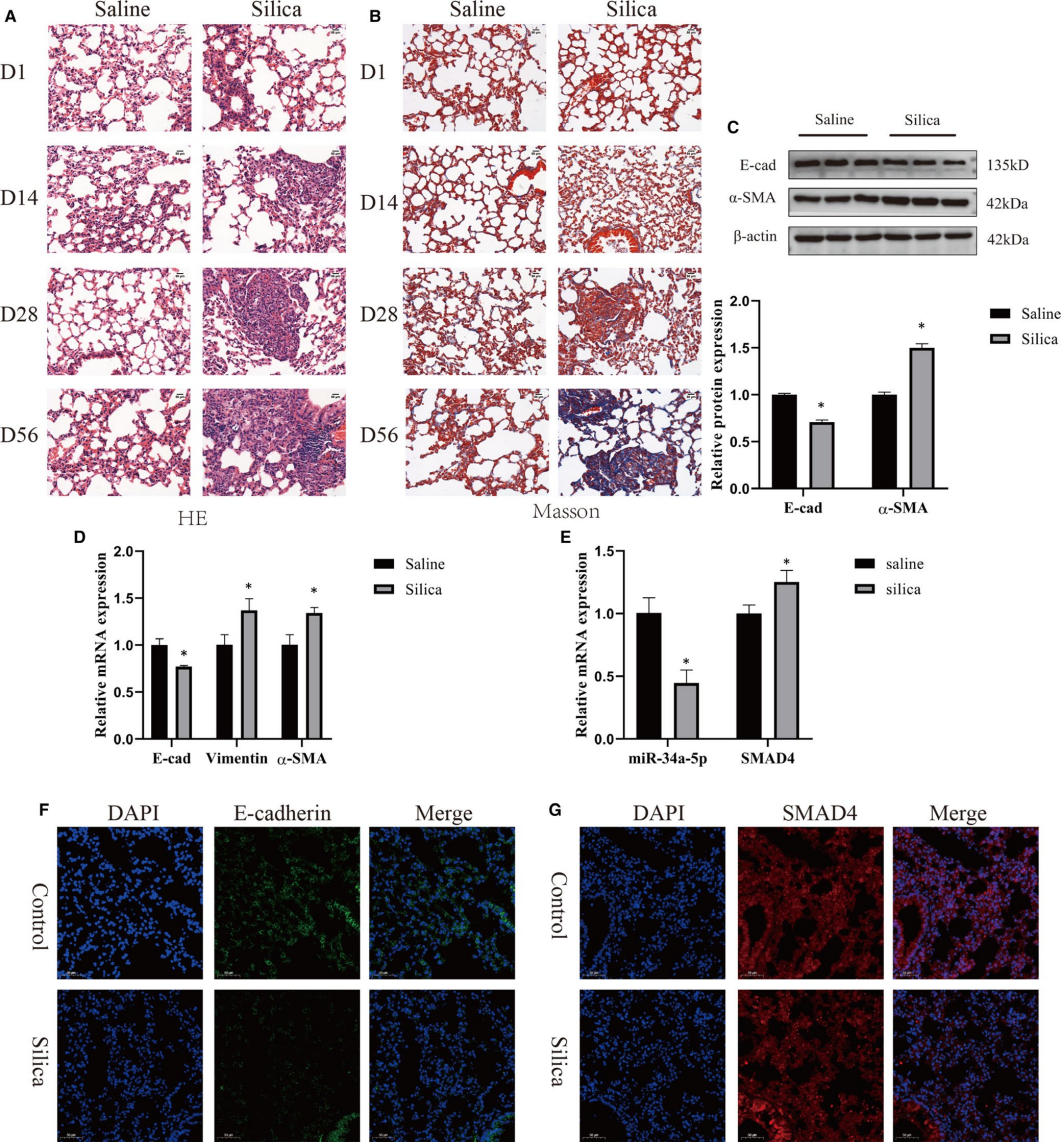
miR‐34a‐5p is down‐regulated, and SMAD4 is up‐regulated in silica‐treated mice. HE (A) and Masson (B) staining of mouse lung tissues show pathological changes (light micrograph magnifications of 200×). The mice were sacrificed at different time points after silica treatment intratracheally (days 1, 14, 28 and 56). Western blotting (C) and qPCR (D) showing E‐cad, VIM and α‐SMA levels in lung tissues at 56 days. qPCR (E)analysis of miR‐34a‐5p and SMAD4 levels in different groups at 56 days. All the data are expressed as means ± SD, and the experiments were replicated at least 3 independently. *P < .05 versus saline group. Immunofluorescence staining of E‐cad (F) and SMAD4 (G) in lung tissues in different groups at 56 days. Scale bar, 50 µm

After silica instillation for 56 days, mouse lungs were harvested. HE staining showed that alveolar wall thickened, alveolar structure destroyed and the numbers of cell nodules increased (Figure 1A). Masson trichrome staining showed that the collagen fibre was deposited in the mice instilled with silica compared with the saline‐treated mice (Figure 1B). The expression of E‐cad significantly decreased, whereas α‐SMA increased in lung tissue both at the protein (Figure 1C, G) and mRNA levels (Figure 1D). Next, the RT‐PCR and immunofluorescence assay all confirmed that SMAD4 was up‐regulated in the silica‐treated group and miR‐34a‐5p was down‐regulated (Figure 1E, F). These results suggested that the silica‐induced pulmonary fibrosis model was established successfully and the expression of miR‐34a‐5p decreased and the expression of SMAD4 increased in this process.

### 3.2 Silencing or overexpression miR‐34a‐5p regulate SMAD4 expression and EMT process in cell model

To explore whether TGF‐β1 could induce the occurrence of EMT in A549 cells, we performed the recognition and detection of TGF‐β1 induced EMT in vitro. The microscope showed that the cell morphology changed from the typical epithelial morphology to spindle fibroblast morphology (Figure S1A). RT‐PCR and Western blot results showed that the expression of E‐cad decreased, while the expression of α‐SMA and vimentin increased (Figure S1B, C). Meanwhile, the expression of miR‐34a‐5p decreased and the expression of SMAD4 increased (*P* < .05) (Figure S1D, E).

**FIGURE 2:**
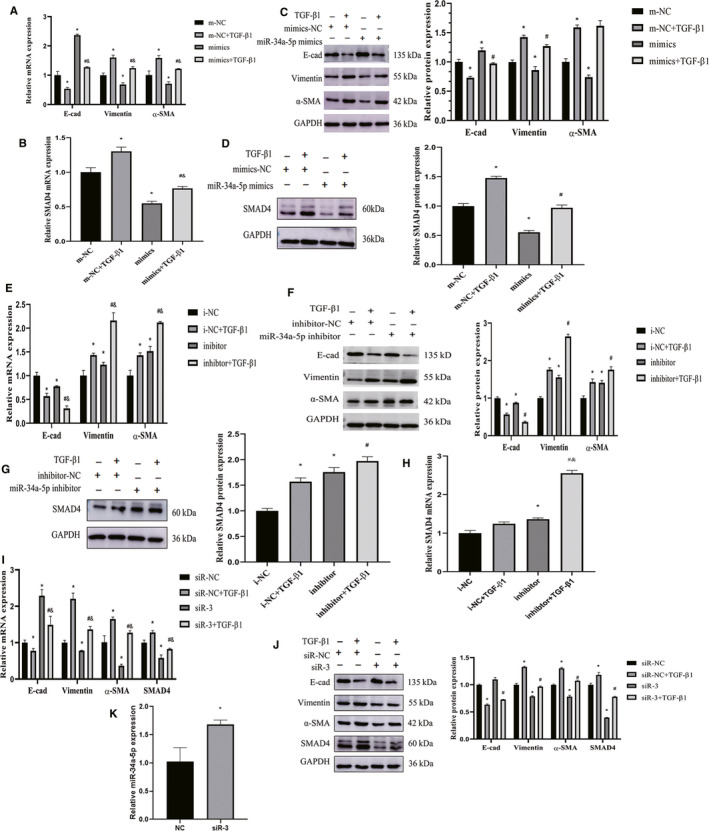
Silencing or overexpression miR‐34a‐5p regulate SMAD4 expression and EMT process in A549 cells. A, qPCR analysis showed that E‐cad, vimentin and α‐SMA mRNA levels in NC, NC + TGF‐β1, mimics, mimics + TGF‐β1 groups. B, PCR analysis showed that SMAD4 mRNA levels in NC, NC + TGF‐β1, mimics, mimics + TGF‐β1 groups. C, Western blot analysis showed that E‐cad, vimentin and α‐SMA protein levels in NC, NC + TGF‐β1, mimics, mimics + TGF‐β1 groups. D, Western blot analysis showed that SMAD4 protein levels in NC, NC + TGF‐β1, mimics, mimics + TGF‐β1 groups. All the data are expressed as means ± SD, and the experiments were replicated at least 3 independently. *P < .05 versus NC group, ^#^ *P* < .05 versus NC + TGF‐β1 group and ^&^ *P* < .05 versus mimics group. E, qPCR was used to analyse the expression of EMT‐related genes such as E‐cad, vimentin, α‐SMA. F, Western blot was used to measure the protein expression of EMT‐related genes, and the quantification analysis was shown as a graph. G, Western blot was used to analyse the expression of SMAD4 in protein level. H, qPCR was used to analyse the expression of SMAD4 in mRNA level. All the data are expressed as means ± SD, and the experiments were replicated at least 3 independently. *P < .05 versus NC group, ^#^ *P* < .05 versus NC + TGF‐β1 group and ^&^ *P* < .05 versus inhibitor group. Knockdown of SMAD4 in A549 cells. I, qPCR was used to analyse the expression of SMAD4 and EMT‐related genes. J, Western blot was used to measure the protein expression of SMAD4 and EMT‐related genes, and the quantification analysis was shown as a graph. K, qPCR analysis showed that transfection siR‐3 slightly increased the expression of miR‐34a‐5p in A549 cells. All the data are expressed as means ± SD, and the experiments were replicated at least 3 independently. *P < .05 versus NC group, ^#^ *P* < .05 versus NC + TGF‐β1 group and ^&^ *P* < .05 versus siR‐3 group

To study the mechanism of miR‐34a‐5p, we detected the efficiency of transfection by the fluorescent‐labelled NC with Laser‐scanning confocal microscopy (Figure S2A). The results showed that the transfection of miR‐34a‐5p mimics remarkably increased the expression level of miR‐34a‐5p (Figure S2B). The miR‐34a‐5p mimics enhanced the expression of E‐cad and down‐regulated vimentin at mRNA (Figure 2A) and protein (Figure 2C) levels. In addition, miR‐34a‐5p mimics decreased the expression of SMAD4 at mRNA (Figure 2B) and protein (Figure 2D) levels. It indicated that the miR‐34a‐5p mimics reduced the SMAD4 expression and impeded EMT process in vitro.

In order to fully elucidate the mechanism of miR‐34a‐5p, we repressed the expression of miR‐34a‐5p by transiently transfecting miR‐34a‐5p inhibitors into A549 cells. RT‐PCR results indicated that the transfection of miR‐34a‐5p inhibitors remarkably decreased the miR‐34a‐5p level (Figure S3A). miR‐34a‐5p inhibitor increased the expression of mesenchymal markers vimentin and α‐SMA, and decreased the expression of epithelial marker E‐cad which was induced by TGF‐β1 (Figure 2E, F). In addition, compared with the TGF‐β1 + miR‐34a‐5p inhibitor NC group, the level of SMAD4 obviously increased in TGF‐β1 + miR‐34a‐5p inhibitor group (Figure 2G, H). It indicated that miR‐34a‐5p inhibitor could aggravate the EMT process in vitro.

### 3.3 miR‐34a‐5p targets SMAD4 to regulate the EMT process

Luciferase reporter revealed that the activity of SMAD4 3’‐UTR was significantly inhibited by miR‐34a‐5p mimics in SMAD4‐WT reporter plasmid but not in SMAD4‐MUT reporter plasmid (Supplementary material, Figure S4A, B). Above data suggested that SMAD4 is a direct target of miR‐34a‐5p.

To further explore whether SMAD4 is a key factor of EMT. Firstly, we detected the efficiency of transfection by the fluorescent‐labelled siR‐NC with laser‐scanning confocal microscopy (Figure S5A). All of the siRNA reduced SMAD4 expression at the mRNA level (Figure S5B), and siR‐3 was selected for subsequent experiments because of its high inhibitory efficiency. The results showed that the SMAD4 were significantly decreased at mRNA and protein levels in the groups which knockdown of SMAD4 via transfection siR‐3(Figure 2I‐J). Moreover, the mesenchymal markers α‐SMA and vimentin significantly decreased in siR‐3 group, but the epithelial marker E‐cad increased (Figure 2I‐J). Interestingly, we found that the expression of miR‐34a‐5p was slightly increased with statistical significant by transfection siR‐3 of SMAD4 (Figure 2K). It indicated that SMAD4 and miR‐34a‐5p form negative feedback involve in EMT process.

## 4 DISCUSSION

Inhalation of silica particles leads to impaired lung function, which is associated with the development of silicosis. However, its mechanism is not fully elucidated, treatment of silicosis still stucks in a bottleneck.^9^ Understanding the complex network regulation mechanism of silicosis contributes to identifying the potential therapeutic targets.

An in‐depth study of EMT contributes to understanding the mechanism of silicosis. Considering that TGF‐β1 could strongly induce EMT,^10^, ^11^ we establish a EMT cell models using TGF‐β1 stimulated A549 cells, and found that the epithelial maker E‐cad has a reduced expression and mesenchymal markers vimentin and α‐SMA have a relatively increased expression which are in line with expectations.

Accumulating evidence suggests that several abnormally expressed miRNAs involved in the process of pulmonary fibrosis play an important regulatory role.^5^, ^12^ In our study, it was verified that overexpression of miR‐34a might partly reverse the process of EMT, whereas down‐regulation miR‐34a was capable of promoting EMT in vitro. All of the findings indicate that miR‐34a probably affects the progress of pulmonary fibrosis by negatively regulating the EMT process. However, in BLM‐induced EMT using RLE/Abca3 or A549/ABCA3, a cell line having alveolar typeⅡcell‐like phenotype, it was found that the expression of miR‐34a was increased.^13^, ^14^ This suggests that miR‐34a may promote pulmonary fibrosis. There were significant differences between these studies, possibly due to the different cell types and the heterogeneity of disease stages.

SMAD4, the only recognized Co‐Smad in mammals,^15^ abnormally affects TGF‐β signalling pathway. Dual‐luciferase reporter assay presented revealed that overexpression miR‐34a suppressed TGF‐β‐induced EMT process in A549 cells by targeting the 3’‐UTR of SMAD4 and down‐regulated its expression both in mRNA and protein expression levels. Furthermore, knockdown of SMAD4 in A549 cells by using siRNA technique attenuated expression of vimentin and α‐SMA. Interestingly, transfection of SMAD4 siRNA significantly increased the expression of miR‐34a. Thus, this meant that miR‐34a functions may through some negative feedback to influence EMT in pulmonary fibrosis in vitro.

In conclusion, our results revealed that miR‐34a could inhibit EMT in pulmonary fibrosis via targeting SMAD4. Thus, a novel strategy may be provided by these findings to prevent and treat silica‐induced pulmonary fibrosis.

## CONFLICT OF INTEREST

No other potential conflict of interest was reported.

## AUTHOR CONTRIBUTIONS

**Yuanmeng Qi:** Conceptualization (equal); Formal analysis (equal); Supervision (equal); Writing‐original draft (equal). **Ahui Zhao:** Visualization (equal); Writing‐review & editing (equal). **Luheng Jin:** Writing‐review & editing (equal). **Peiyan Yang:** Resources (equal); Visualization (equal). **Changfu Hao:** Conceptualization (equal); Data curation (equal); Visualization (equal).

## Supporting information

Fig S1

Fig S2

Fig S3

Fig S4

Fig S5

Fig S6

Supplementary Material

## Data Availability

All data and model generated or used during the study appear in the submitted article.
