# Supplementary figures and images for: miR‐34a‐5p Attenuates EMT through targeting SMAD4 in silica‐induced pulmonary fibrosis
Source: J Cell Mol Med. 2020 Sep 14;24(20):12219–24. doi: 10.1111/jcmm.15853 (PMC7579717; doi:10.1111/jcmm.15853)

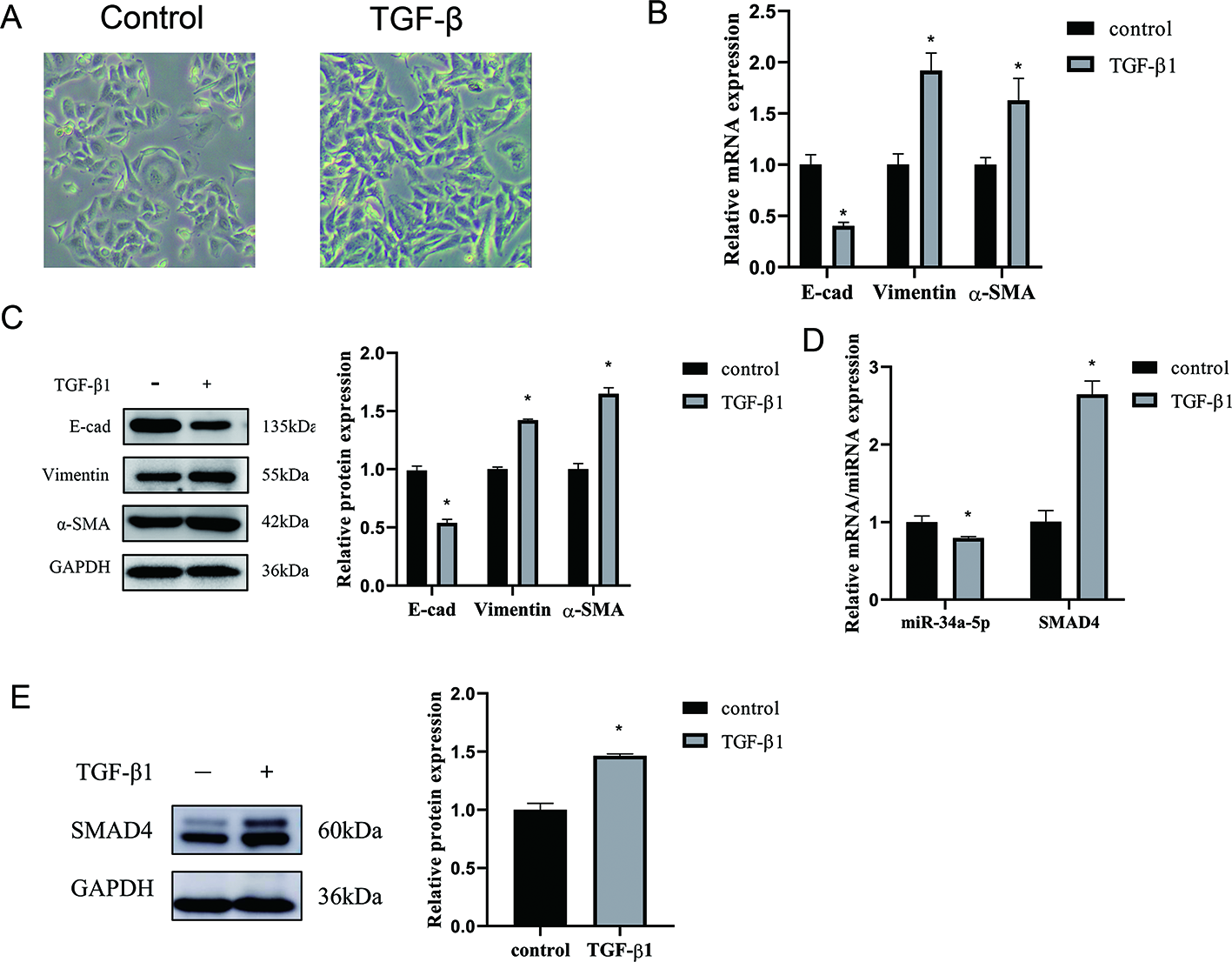

Supplement: Supplementary file 1 — Fig S1 [file JCMM-24-12219-s001.tif]

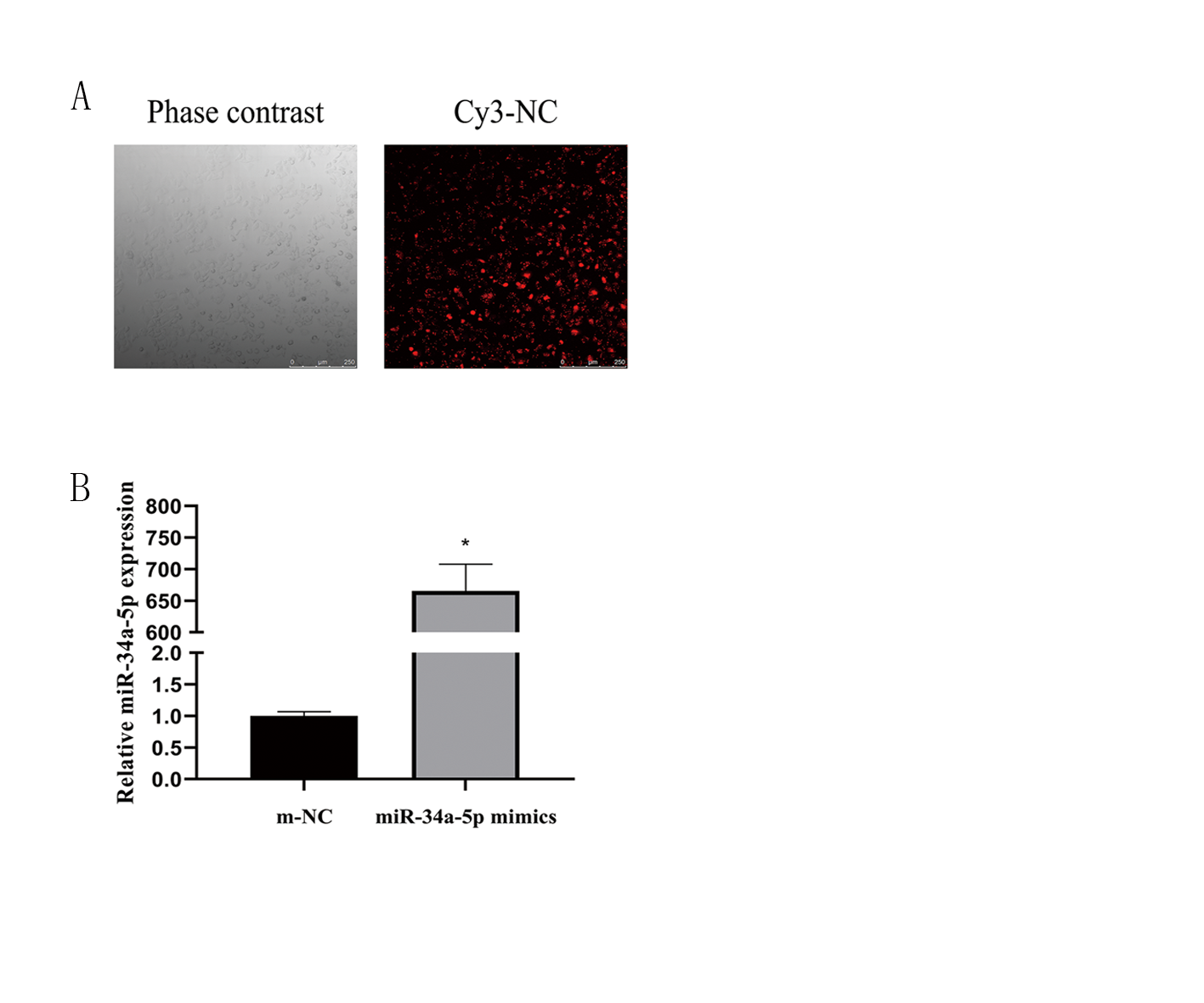

Supplement: Supplementary file 2 — Fig S2 [file JCMM-24-12219-s002.tif]

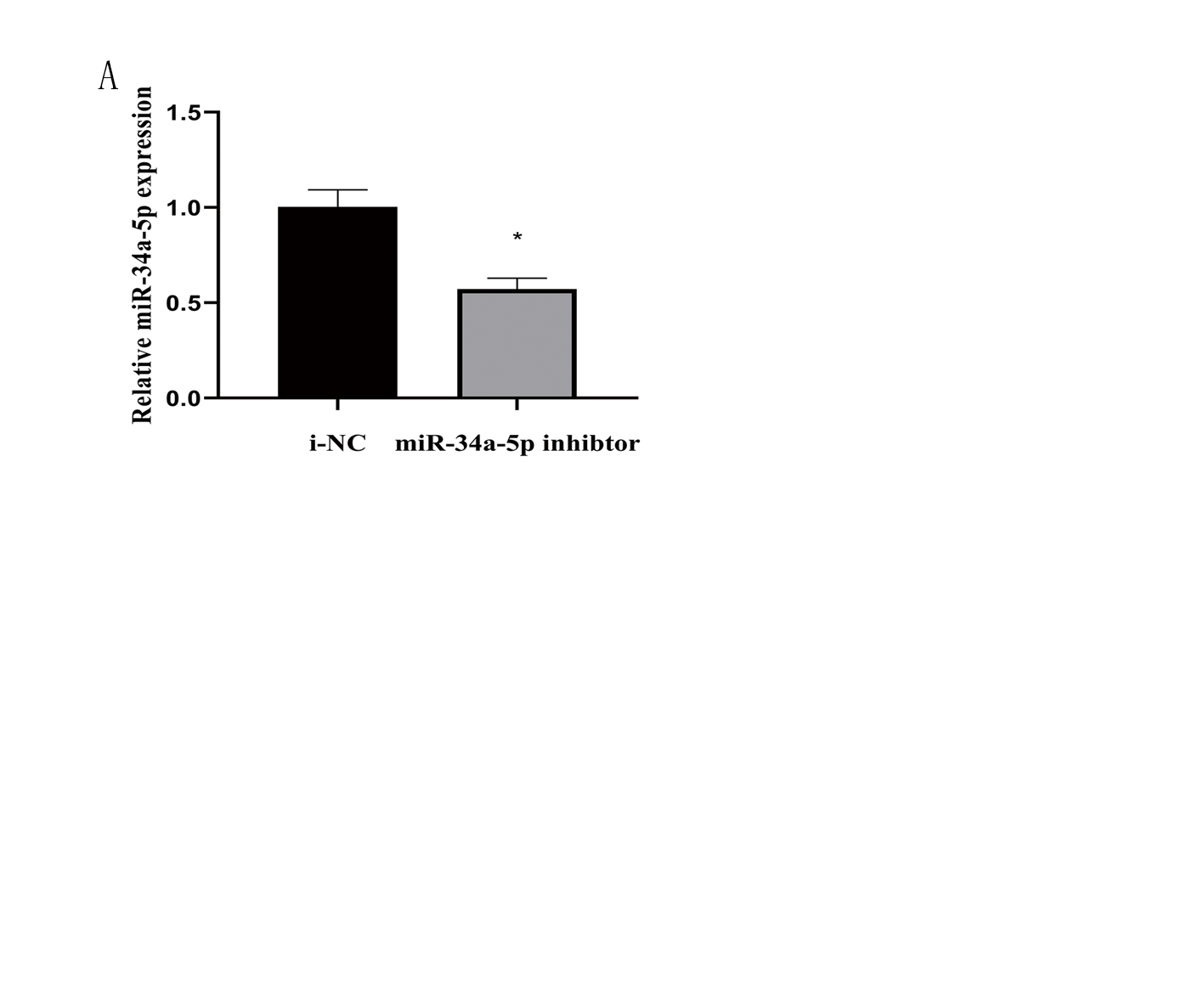

Supplement: Supplementary file 3 — Fig S3 [file JCMM-24-12219-s003.tif]

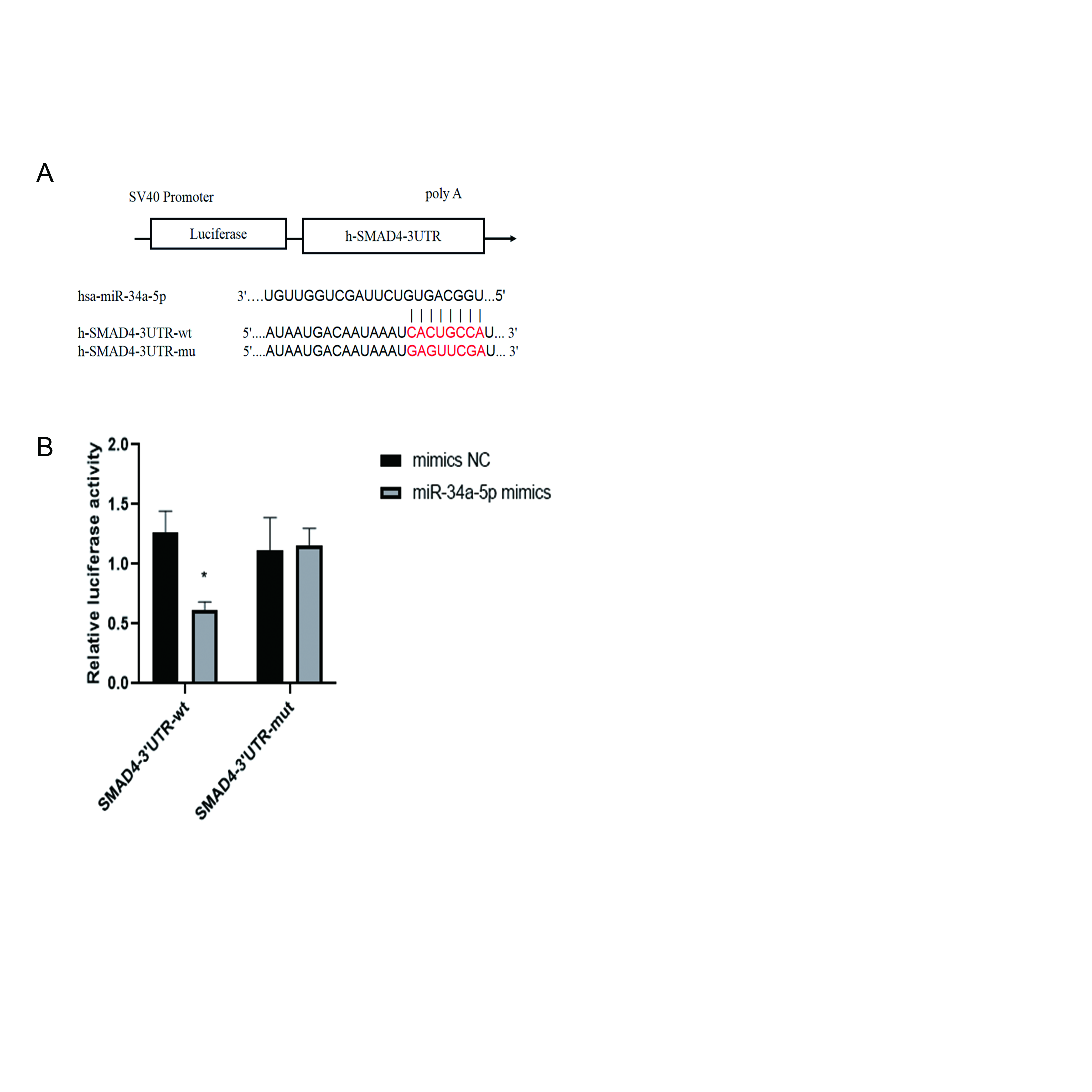

Supplement: Supplementary file 4 — Fig S4 [file JCMM-24-12219-s004.tif]

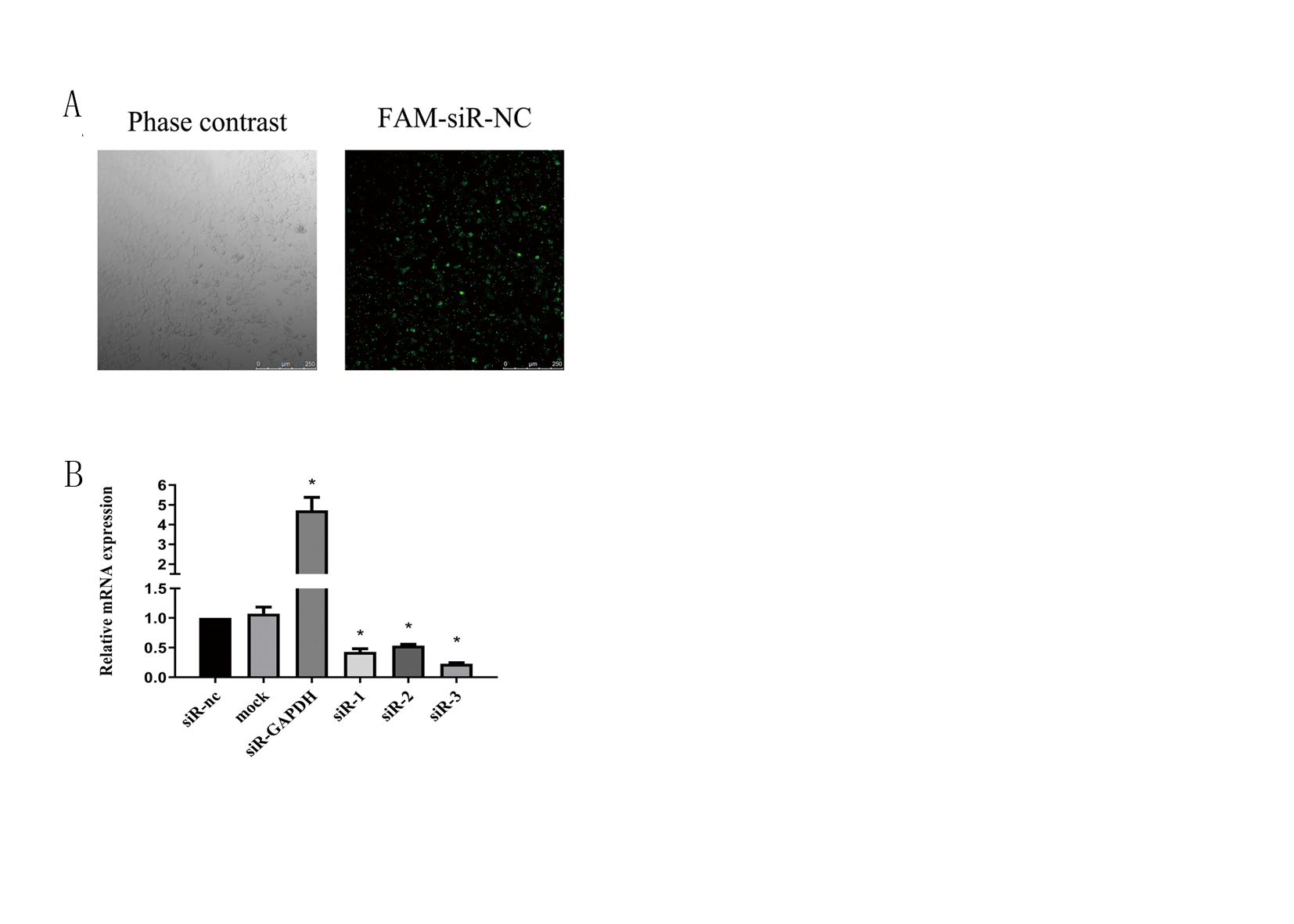

Supplement: Supplementary file 5 — Fig S5 [file JCMM-24-12219-s005.tif]

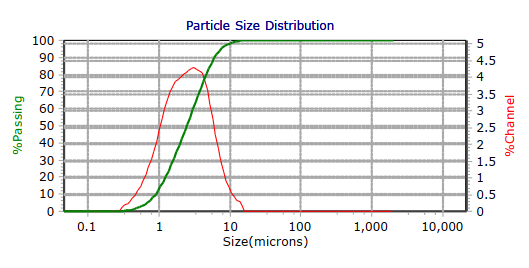

Supplement: Supplementary file 6 — Fig S6 [file JCMM-24-12219-s006.tif]
