# Supplementary material for: miR‐34a‐5p Attenuates EMT through targeting SMAD4 in silica‐induced pulmonary fibrosis
Source: J Cell Mol Med. 2020 Sep 14;24(20):12219–24. doi: 10.1111/jcmm.15853 (PMC7579717; doi:10.1111/jcmm.15853)
Supplement: Supplementary file 7 — Supplementary Material [file JCMM-24-12219-s007.docx]

**Silica particles**

Silica particles, of which content was >99% and average particles size was 0.5-10 μm，were purchased from sigma-Aldrich (Shanghai, China). We measured the silica particles size distribution by Microtrac S3000 laser particle size analyzer (Microtrac Inc, USA). The particle size distribution supplement shown in figure 1. The silica particles were ground and sterilized before used in cell and animal experiements.

**Euthanization produce**

The mice were sacrificed with a sealed carbon dioxide (CO2) euthanasia device at different time points of experiment. To minimize the pain of killing the mice, we put a certain amount of CO2 into the euthanization device before we put them in. In addition, after the mice were euthanatized, we keep pumping in CO2 for two minutes.

**Histopathology analysis**

10% formalin treated right lower lung of mice for at least 48 hours. After that, the lung tissues were embedded in paraffin and then were cut into 5um thick sections. The lung tissue sections were stained with hematoxylin, eosin (H & E) and Masson trichrome staining to evaluate the degree of pulmonary fibrosis. The samples were observed with microscope in 200x magnifications.

**Cell cultures**

A549 cells (human lung type Ⅱ alveolar epithelial adenocarcinoma cell lines) were purchased from the cell bank of the Chinese academy of sciences (Shanghai, China). A549 cells were grown in RPMI medium (Hyclone, south Logan, USA) added with10% fetal bovine serum (FBS) (Hyclone, south Logan, USA). The cells were cultured at 37℃ in a humidified incubator with an atmosphere of 5% CO_2_ and 95% air. Approximately 3×10^5^ cells were Inoculated in the six-well plates, and then cultured in incubator for 24h to allow to adhere the wall. When A549 cells were cultured to 80% confluence, they were stimulated by 5ng/mL human TGF-β1 (PeroTech, NJ, USA). After 48h, these cells were harvested for further experiments.

**Cell transfection**

Mimic control (mimic NC), miR-34a-5p mimics, siRNA-smad4, inhibitor control and miR-34a-5p inhibitor were synthesized by GenePharma Co (Shanghai, China). A549 cells were transfected with lipofectamine^®^ 3000 transfection reagent (Invitrogen, USA) carried out according to the reagent’t instructions. Before transfection, approximately 3×10^5^ cells were grown in the six-well plates for 18-24 h. 150 uL serum-free medium containing 50nM mimics or mimics NC, 100 nM inhibitor or inhibitor NC, 50 nM siRNA-smad4 or siRNA-NC was mixed with another 150 uL serum-free medium containing 5 uL transfection reagent. The mixture was incubated at the room temperature for 10 minutes. Then they were transferred to each well of six-weel plates, after for 6h at 37℃in incubator, the medium was replaced with or without TGF-β1 complete medium and the cells were harvested for further study after 48 h. Meanwhile, the sequences are as follows: the hsa miR-34a-5p mimics sense is 5’-UGGCAGUGUCUUAGCUGGUUGU-3’ and the antisense is 5’-AACCAGCUAAGACACUGCCAUU-3’; miR-34a-5p inhibitor sense is 5’-ACAACCAGCUAAGACACUGCCA-3’; siRNA-smad4 sense is 5’-GCUCCUAGACGAAGUACUUTT-3’ and the antisense is 5’-AAGUACUUCGUCUAGGAGCTT-3’.

**qRT-PCR analysis**

Total RNA obtained from lung tissues and cells was extracted using RNAiso Plus (TaKaRa) according to the manufacturer protocol. Mir-X miRNA Reagent Kit (TaKaRa) and PrimerScriptTM RT Reagent Kit (TaKaRa) were used to reverse-transcribed miRNA and mRNA respectively according to the manufacturer protocol. The fold changes of gene expression were calculated using the 2^-△△CT^ method by normalizing to glyceraldehyde-phosphate dehydrogenase(GAPDH) or U6. For statistical analysis of mRNA and miRNA expression levels, at least three times biological replicates were performed in the study. All the sequences of Primers used in the experiment are given in Table1.

Table1 Primers used for relative mRNA/miRNA level analysis by qRT-PCR.

| mRNA/microRNA | Sequence(5’-3’) |
| --- | --- |
| Ms/hsa miR-34a-5p-F | GTGGCAGTGTCTTAGCTGGTTGT |
| Ms/hsa miR-34a-5p-R | (Tokara, Tokyo, Japan) |
| Hsa-GAPDH-F | ACCCAGAAGACTGTGGATGG |
| Hsa-GAPDH-R | TCTAGACGGCAGGTCAGGT |
| Hsa-Ecad-F | AGGATGACACCCGGGACAAC |
| Hsa-Ecad-R | TGCAGCTGGCTCAAGTCAAAG |
| Hsa-Vimentin-F | CCTTGAACGCAAAGTGGAATC |
| Hsa-Vimentin-R | GACATGCTGTTCCTGAATCTGAG |
| Hsa-α-Sma-F | ATTGCCGACCGAATGCAGA |
| Hsa-α-Sma-R | ATGGAGCCACCGATCCAGAC |
| Hsa-Smad4-F | ACGAACGAGTTGTATCACCTGG |
| Hsa-Smad4-R | TGCACGATTACTTGGTGGATG |
| Ms-GAPDH-F | GTGAAGCAGGCATCTGAGGG |
| Ms-GAPDH-R | CGAAGGTGGAAGAGTGGGAGT |
| Ms-E-cad-F | CCTGTCTTCAACCCAAGCAC |
| Ms-E-cad-R | CAACAACGAACTGCTGGTCA |
| Ms-Vimentin-F | CGGCTGCGAGAGAAATTGC |
| Ms-Vimentin-R | CCACTTTCCGTTCAAGGTCAAG |
| Ms-α-SMA-F | GAGCATCCGACACTGCTGAC |
| Ms-α-SMA-R | GCACAGCCTGAATAGCCACA |
| Ms-samd4-F | AGGTGGCCTGATCTACACAAG |
| Ms-samd4-R | ACCCGCTCATAGTGATATGGATT |

Ms, mouse; Hsa, homo sapiens

**Western blot**

The protein extractions obtained from lung tissues and cells were extracted using RIPA lysis buffer (Boster Biological Technology) containing Phenylmethanesulfonyl fluoride (PMSF; Boster Biological Technology). Various proteins were separated in 10% SDS-PAGE polyacrylamide gradient gels and transferred to PVDF membranes (Boster Biological Technology). subsequently, the PVDF membranes were blocked in 5% nonfat dry milk for 2h at room temperature and incubated with primary antibodies at 4℃ overnight. After washing three times with TBST, the membranes were incubated with the secondary antibody for 1h at room temperature. The stripe of protein was detected with ECL detection reagent (Cwbiotech). Image J software was used to quantify the target proteins.

The antibodies used in this experiment were as follows: anti-E-cad（Cell Signaling Technology), anti-Vimentin (Cell Signaling Technology), anti-α-SMA（Cell Signaling Technology), anti-SMAD4 (Cell Signaling Technology) and anti-GAPDH (Cell Signaling Technology). HRP-conjugated Affinipure Goat Anti-Rabbit IgG (proteintech) was used as the secondary antibody.

**Dual luciferase reported gene assays**

The wild type (Wt) of SMAD4, which contains miR-34a-5p binding sites of 3’-UTR region,and its mutant (Mut) sequence were conducted and cloned into the pSI-Check2 plasmid (Hanbio Biotechnology). The A549 cells were seeded in 96-well plates for 24h , and then co-transfected with 200 ng Wt or Mut luciferase reporter plasmids of SMAD4 and 100 nM miR-34a-5p mimics or mimics NC using Lipofectamine® 3000 (Invitrogen). After 48h, luciferase activities were determined by Luciferase Assay Reagent (Progema).

[**Immunofluorescence**](G:/%E6%9C%89%E9%81%93%E7%BF%BB%E8%AF%91/Dict/8.5.3.0/resultui/html/index.html#/javascript:;) **assay**

After being treated with deparaffinization, rehydration and antigen retrieval, paraffin-embedded lung tissue washed with PBS. Then it was fixed 0.5% Triton X-100 for 30 min, and blocked with 5% BSA for 1h. Slides were incubated with anti-E-cad antibody (Cell Signaling Technology), anti-SMAD4 (Cell Signaling Technology) at 4℃ overnight. After being washed three times for 5 min with PBS, it was incubated with secondary antibodies labeled with fluorescein isothiocyanate (FITC) for 1h at room temperature away from light. Subsequently, it was incubated with DAPI for nuclear staining away from light. At last, the images were acquired through confocal laser scanning microscopy (LSCM).

**Statistical analysis**

All the data were expressed as Mean ± SD and the statistical analyze were calculated with SPSS 21.0 software. The comparison of two groups was analysed by Independent-samples *t test*, whereas the comparison between more groups was analysed by one-way analysis of variance (ANOVA). *P*<0.05 was defined as statistically significant.
